# Mistaken ST-Elevation Myocardial Infarction

**DOI:** 10.5811/westjem.2015.9.28631

**Published:** 2015-11-12

**Authors:** Brian J. Wolk

**Affiliations:** * Loma Linda University School of Medicine, Department of Emergency Medicine, Loma Linda, California

A 66-year-old female was transferred from an outside hospital for possible ST segment elevation myocardial infarction (STEMI). The patient reported feeling poorly for the last day, with epigastric pain, nausea, and multiple episodes of vomiting. Patient’s medical history was significant for diabetes mellitus, hypertension, atrial fibrillation, and multiple sclerosis. Electrocardiogram (EKG) was as noted (Figure). Initial troponin was 0.14 (<0.03ng/mL). The patient was taken emergently to the cardiac cath lab for possible posterior STEMI. Angiogram demonstrated no significant evidence of coronary artery disease, with an EF of 75%.

Digoxin concentration subsequently returned at 8.8ng/mL (reference range 0.5–1ng/mL). The ST segment changes gradually improved as the digoxin concentration declined. An echocardiogram demonstrated moderate concentric left-ventricular hypertrophy with estimated ejection fraction of 80%, rheumatic heart disease, and possible hypertrophic obstructive cardiomyopathy physiology. Troponin peaked at 0.29ng/mL and then returned to baseline. Creatine kinase remained within normal limits.

## DISCUSSION

Digoxin may cause a multitude of EKG changes including ST depression and numerous cardiac dysrhythmias. 1,2 Differentiation of ST depression in patients with ischemic heart disease and digoxin presence may be feasible in patients undergoing stress testing using heart rate analysis, 2 but the critical nature of a potential acute myocardial infarction patient likely prohibits this in-depth analysis. ST depression may appear indistinguishable from ischemic changes, and the history of digoxin use or digoxin concentration testing should be considered in a patient with nausea and vomiting and signs or symptoms of acute coronary syndrome with marked ST depression.

## Figures and Tables

**Figure:**
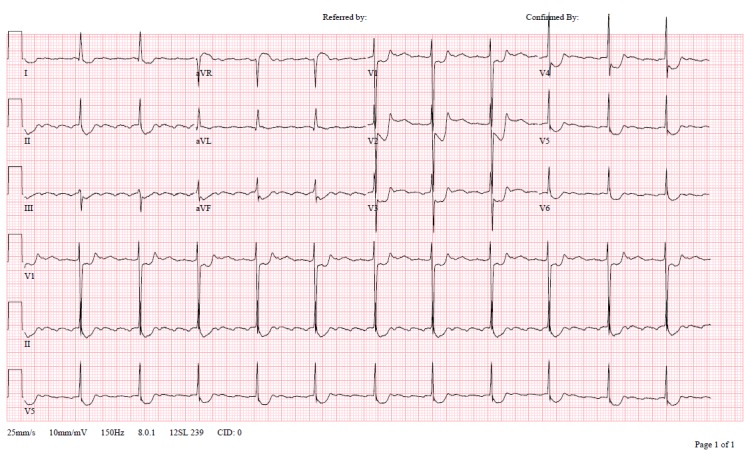
Initial electrocardiogram of patient with elevated digoxin concentration
